# TAR RNA Mimicry of INI1 and Its Influence on Non-Integration Function of HIV-1 Integrase

**DOI:** 10.3390/v17050693

**Published:** 2025-05-11

**Authors:** Ganjam V. Kalpana, Emilie Ernst, Swati Haldar

**Affiliations:** Department of Genetics, Albert Einstein College of Medicine, Bronx, NY 10461, USA

**Keywords:** HIV-1, integrase, INI1/SMARCB1, TAR RNA, particle morphogenesis, protein–RNA mimicry, assembly

## Abstract

HIV-1 integrase (IN), an essential viral protein that catalyzes integration, also influences non-integration functions such as particle production and morphogenesis. The mechanism by which non-integration functions are mediated is not completely understood. Several factors influence these non-integration functions, including the ability of IN to bind to viral RNA. INI1 is an integrase-binding host factor that influences HIV-1 replication at multiple stages, including particle production and particle morphogenesis. IN mutants defective for binding to INI1 are also defective for particle morphogenesis, similar to RNA-binding-defective IN mutants. Studies have indicated that the highly conserved Repeat (Rpt) 1, the IN-binding domain of INI1, structurally mimics TAR RNA, and that Rpt1 and TAR RNA compete for binding to IN. Based on the RNA mimicry, we propose that INI1 may function as a “place-holder” for viral RNA to facilitate proper ribonucleoprotein complex formation required during the assembly and particle morphogenesis of the HIV-1 virus. These studies suggest that drugs that target IN/INI1 interaction may lead to dual inhibition of both IN/INI1 and IN/RNA interactions to curb HIV-1 replication.

## 1. Introduction

Approximately 39 million people worldwide are living with human immunodeficiency virus or HIV-1, the causative agent of AIDS [1]. Despite decades of research, HIV has no known cure (except in a few cases) or available vaccine [2,3]. Current anti-retroviral therapy (ART) is effective, but it causes many side effects and drug-resistant mutations [4,5,6]. Furthermore, the presence of HIV-1 latent reservoirs makes it difficult to eliminate AIDS, necessitating the development of novel therapeutics [7]. Most current anti-HIV drugs target viral proteins at various stages of viral replication rather than host–virus interactions [8]. Although many essential host-virus protein–protein interactions (PPIs) have been uncovered, only a few are targeted by FDA-approved drugs (e.g., Maraviroc) [9]. Targeting essential host–virus interactions, rather than viral proteins, has an advantage as it may be harder for the virus to develop resistance to such drugs. Drug-resistant mutations in the virus, while preventing the viral protein from binding to the drug, may also inhibit interaction with the host factor, making the virus defective for replication. A broader understanding of host–virus interactions and their interfaces is needed to develop new classes of drugs that target these interactions.

Integrase (IN) is an essential virus-encoded enzyme that catalyzes the integration of viral DNA into the host genome, and several integrase inhibitors are part of anti-retroviral regimens [8,10]. In addition to integration, IN has been shown to influence stages other than integration, such as reverse transcription, particle production, and particle morphogenesis [10,11,12,13,14]. The mechanism by which IN influences non-integration function is not completely understood. However, it is of interest as studies have suggested that these functions can be targeted to inhibit HIV-1 replication. An earlier study suggested that the non-integration functions could be influenced by host factors that interact with IN. For example, a dominant negative mutant of the IN-interacting host factor, INI1, inhibited HIV-1 late events in a manner dependent on its ability to bind to IN [15]. Recent studies indicate that the late events could be influenced by a variety of factors that affect IN in some way. Mutations and drugs that lead to aberrant IN multimerization, lack of binding to INI1, or lack of binding to viral RNA all result in defective particle morphology and inhibit the infectivity of the particles produced. [13,15,16,17].

IN interacts with many essential host proteins, including LEDGF [18,19,20] and INI1 (also known as hSNF5, SMARCB1, and BAF47) [15,21]. Extensive studies have established the role of LEDGF in targeting viral integrations into chromatin regions [19,22,23,24,25]. While INI1 is the first host factor to be identified as a binding partner for HIV-1 IN, not much is known about how it influences viral replication, in part due to the lack of understanding of the non-integration functions of IN and the lack of structural information for the IN/INI1 complex [15,21].

The current review article focuses on the studies on the role of INI1 in HIV-1 replication and subsequent progress to utilize IN/INI1 interaction as a potential therapeutic target for developing a novel class of α-HIV-1 inhibitors. Several studies have indicated that IN/INI1 interaction is essential for HIV-1 replication [15,26,27,28,29,30]. These studies have shown that INI1 plays a role in late events of HIV-1 replication, influencing the non-integration function of IN, namely particle production and particle morphogenesis [15,29,30,31]. Our recent discovery that the IN-binding domain of INI1 structurally mimics viral TAR RNA suggests a possible mechanism of its involvement in late events [32]. In this review article, we provide a summary of what is known about the role of INI1 in HIV-1 replication, describe the exciting discovery of RNA mimicry of the IN-binding domain of INI1, and offer a brief recommendation for the future development of drugs that target IN/INI1 interactions to inhibit HIV-1 replication.

## 2. Relevant Sections

### 2.1. Integrase as a Target for Inhibiting HIV-1 Late Events

**Figure 1:**
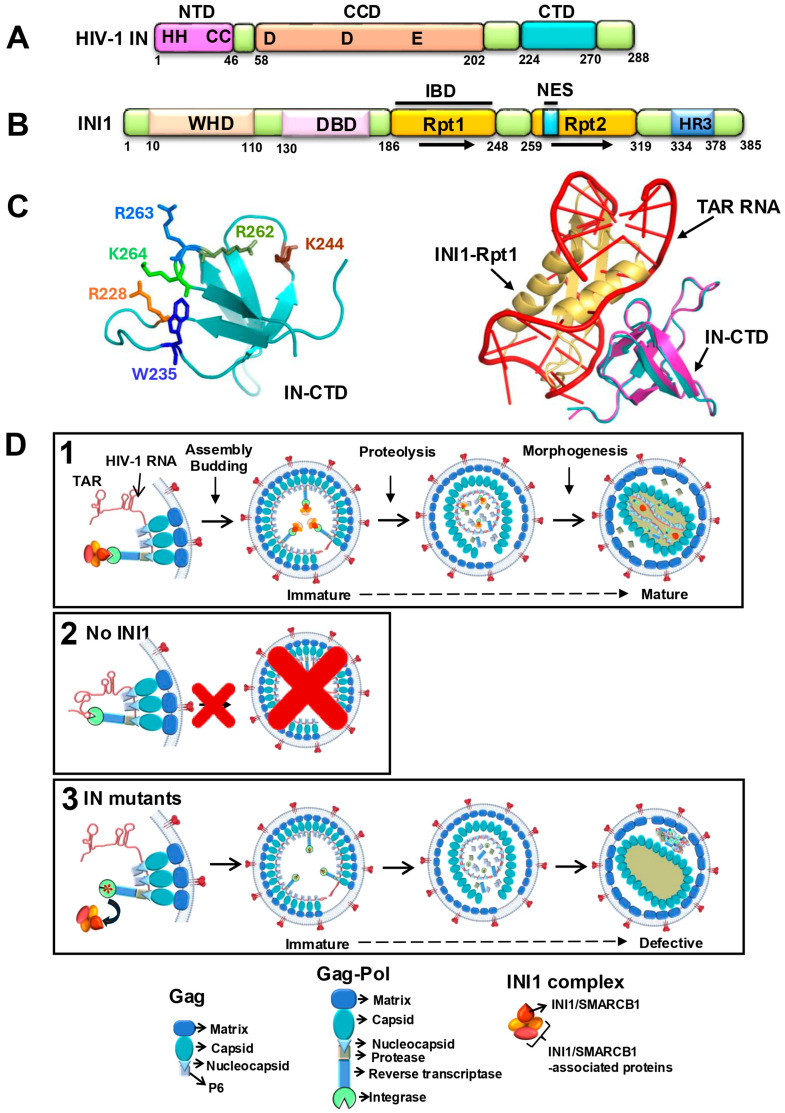
A working model to explain the role of INI1 in HIV-1 replication based on its RNA mimicry: (**A**) A cartoon representing the domain organization of IN. Numbers below the cartoon represent amino acid numbers. The three domains, the N-terminal domain (NTD) with conserved zinc finger residues HHCC, the central core domain (CCD) with conserved DDE residues, and the C-terminal domain (CTD), are indicated. (**B**) A cartoon representing the domain organization of INI1. Numbers below the cartoon represent amino acid numbers. WHD = Winged-Helix DNA-binding domain; DBD = DNA-binding domain; Rpt1 = Repeat 1; Rpt2 = Repeat 2; HR3 = homology region III (also called coiled coil domain); IBD = integrase-binding domain; NES = nuclear export signal (aa 263–276). (**C**) **Left panel**: Structure of IN-CTD (Teal) showing the residues that are involved in binding to both INI1-Rpt1 and TAR. **Right panel**: Superimposed three-dimensional structural models of INI1-Rpt1 (Gold)/IN-CTD (Magenta) complex with TAR RNA (Red)/ IN-CTD (Teal) complex. Rpt1 and TAR RNA fit into each other in three-dimensional space in binding to IN. (**D**) A model to explain the role of TAR RNA mimicry of INI1-Rpt1 domain during HIV-1 assembly. Note: The stoichiometry between GagPol-IN:INI1 is not known, and for the sake of simplicity, a 1:1 ratio is used in this figure. **Panel 1**: In a WT producer cell, INI1 acts as a place-holder and binds to the IN portion of Gag-Pol to prevent RNA binding to it, avoiding steric hindrance. Both RNA and INI1 are incorporated into the virions, resulting in correct particle morphogenesis. **Panel 2**: A lack of INI1 leads to the binding of RNA to the IN portion of Gag-Pol, resulting in defective assembly and particle production. **Panel 3:** RNA-interaction-defective and INI1-interaction-defective mutants of IN (with red asterisk) are impaired for binding to both RNA and INI1, and hence there is no steric hindrance for assembling Gag-Pol. RNA is incorporated into the virions through its binding to NC. However, the lack of binding to IN leads to morphologically defective particles. The bottom panel below panel 3 indicates representative units within Gag, Gag-Pol, and INI1 complexes used for (**D**).

The main function of IN is to catalyze the integration of reverse-transcribed viral DNA into the host chromosome [33]. Integration is a three-step process involving 3′ processing, strand transfer, and the repair of the integration intermediates [13,33]. IN has three domains, an N-terminal zinc finger domain (NTD), a central core domain (CCD), and a C-terminal domain (CTD) (Figure 1A). While the CCD, with its three conserved catalytic residues (D, D, E), catalyzes 3′ processing and strand transfer, the NTD and CTD are engaged in other essential functions required for the reaction. However, many studies have indicated that IN plays a role in events other than integration, including reverse transcription and late events [10,34,35]. These studies have established that certain mutations of IN exhibit “pleiotropic effects” and inhibit non-integration events such as reverse transcription, particle maturation, and/or virion morphogenesis [10,34,35]. These mutants have been classified as Class II IN mutants to distinguish them from those that only affect integration (Class I) and have been well described elsewhere [13,34]. Many of these class II IN mutants produce morphologically defective virions that exhibit electron-dense condensate located eccentrically outside the capsid lattice [13,34]. The mechanism by which IN mutants induce this defective morphology is not understood [14]. Many of these mutants are in the CTD. Interestingly, the CTD has been shown to interact with (i) viral and target DNA; (ii) viral RNA; (iii) other IN domains within the IN tetramer; and iv) other viral and host proteins, including reverse transcriptase, transportin (TRN-SR2), and INI1 [13,32,36]. It is interesting to note that some of these properties of the CTD may influence the non-integration function of IN.

The influence of IN on assembly and particle production can be explained by its biogenesis and presence as a part of the assembling polyproteins. IN is synthesized as part of the Gag-Pol polyprotein, which is expressed at ~20-fold less abundance compared to the Gag polyprotein [37,38]. Gag-Pol consists of subunits of Gag, including matrix (MA), capsid (CA), nucleocapsid (NC), and P6, and the Pol portion consists of the three enzymes, protease (PR), reverse transcriptase (RT), and IN [37]. Both Gag and Gag-Pol are assembled along with viral RNA to form immature virions. Proteolytic cleavage of these polyproteins to produce individual components and their subsequent arrangement during particle maturation leads to the formation of HIV-1 virions with distinct morphology (see Figure 1D, Panel 1) [14,37]. Within the mature HIV-1 virions, CA is arranged in the form of a lattice, forming the typical cone-shaped morphology of the capsid core that encloses an electron-dense ribonucleoprotein complex [14,37]. After processing, the cleaved IN and RT are incorporated within the capsid core along with the viral genomic RNA [14,37]. While the role of Gag is well studied during assembly, maturation, and particle morphogenesis, the role of Gag-Pol in these processes is not well understood. Gag-Pol brings the essential enzymes into the virions for the subsequent function in the target cells and may or may not play a direct role in assembly processes.

A defect in virion particle morphology is also observed: (i) upon treatment of HIV-1 producer cells with allosteric inhibitors of IN (ALLINIs) [39]; (ii) in IN mutants defective for binding to the host factor INI1 [31]; (iii) in IN mutants that cause aberrant multimerization [16]; and (iv) in IN mutants that are defective for binding to viral RNA (vRNA) [17]. It has now been established that defective particle morphogenesis caused by three of the above, namely, ALLINIs, some class II IN mutants, and multimerization-defective mutants, is due to a defect in the ability of IN to bind to viral RNA [11]. Another factor that influences particle morphogenesis is the ability of IN to bind to INI1 [31,32]. The following sections will provide a summary of observations about the influence of the host factor INI1 and how it influences HIV-1 late events and particle morphology.

### 2.2. INI1 Is an IN-Binding Host Factor Essential for Viral Late Events

INI1 is the first HIV-1 IN-interacting host factor identified using a yeast two-hybrid system by screening a human cDNA library against HIV-1 IN as a bait [21]. It is a component of the human SWI/SNF or BAF complex, a multiprotein prototypical ATP-dependent chromatin remodeling complex involved in epigenetic regulation, transcription, and other cellular processes [40,41,42]. INI1 is also a tumor suppressor that is biallelically deleted and/or mutated in many human cancers, including aggressive pediatric rhabdoid tumors and other malignancies [43,44,45]. Based on the role of INI1 in chromatin remodeling, an “integration targeting” hypothesis was proposed for the first time [21,46]. It was suggested that the interaction of this host protein with integrase may lead to the targeting of integration into transcriptionally active and open chromatin regions [21]. Some of the in vitro studies do support the role of INI1 in integration [47]. However, later studies suggested that INI1 also influences non-integration function (see below).

Our laboratory has been studying the role of INI1 since its discovery and has contributed to deciphering INI1 structure–function activities and its role in HIV-1 replication and the mechanism of tumor suppression [15,26,27,28,29,30,31,32,48,49,50,51,52,53,54]. Structure–function studies have indicated that INI1 has two phylogenetically conserved imperfect repeat domains, namely Rpt1 (aa 183–248) and Rpt2 (aa 259–319), connected by a linker region (aa 249–258), and a third conserved domain, the C-terminal homology region III (HR3), with a coiled coil domain (Figure 1B) [53,55]. An N-terminal Winged-Helix DNA-binding domain (WHD) in INI1 has also been identified [56] (Figure 1B). We have demonstrated that Rpt1, but not Rpt2, is necessary and sufficient to bind to HIV-1 IN [57]. The Rpt domains are also involved in protein–protein interactions with various viral and cellular proteins [28,48,58,59]. Furthermore, it was demonstrated that the Rpt1 domain of INI1 binds to the core and C-terminal domains of IN [32,53].

INI1 is a nuclear protein [49]. However, a masked exportin 1-dependent nuclear export signal (NES) in this protein has been identified that allows it to shuttle to the cytoplasm (Figure 1B) [30,32,49]. In the cytoplasm, INI1 binds to IN within the context of Gag-Pol and is incorporated into HIV-1 virions [15,30]. Intriguingly, it appears that other components of the SWI/SNF complex, including BRG1, BRM, BAF155, and BAF170, are not incorporated into virions along with INI1 [28]. Furthermore, it was demonstrated that INI1 can interact with SAP18 and recruit some of the components of the Sin3A/SAP18 complex into HIV-1 virions [28]. Several studies from our laboratory indicate that INI1 is a linchpin for HIV-1 assembly and particle production [15,26,27,29,30,32]. One of these studies, which was reported in 2001, indicated that INI1 influences HIV-1 late events, for the first time [15]. In this study, it was demonstrated that a fragment of INI1 containing Rpt1, termed S6 (= INI1_183–294_ = Rpt1 + linker + part of Rpt2), binds to IN within the context of Gag-Pol and that this fragment, when expressed in the producer cells, inhibits HIV-1 particle production up to 4–5 logs in a dominant negative manner [15]. Mutants of IN that were defective for binding to INI1 (H12N) were not inhibited by S6, and mutants of S6 (E3, D225G) that were defective for binding to IN inhibited HIV-1 particle production to a much lesser extent, indicating that protein–protein interaction between S6 and HIV-1 IN was required for inhibitory effects [15]. Furthermore, the inhibitory effects of S6 were dramatically reduced in trans-complementation assays, where IN was removed from the context of Gag-Pol and expressed in *trans* as Vpr-IN, indicating that the inhibitory effects of S6 were mediated through its binding to IN within the context of Gag-Pol [15]. In addition, the trans-dominant effect of S6 was specific to HIV-1 IN, and the particle production of other related lentiviruses, including SIV and HIV-2 were not inhibited by S6 [30]. The ability of S6 to inhibit HIV-1 late events was correlated to the selectivity of binding of INI1 to HIV-1 IN and the lack of its binding to integrases from other related lentiviruses [30]. These studies strongly suggested that the trans-dominant negative mutant S6 inhibited HIV-1 assembly and particle production by sequestering Gag-Pol through direct binding, preventing its binding to full-length INI1. These studies, for the first time, shed light on the possibility that the pleiotropic effects exhibited by IN mutants could be due to the involvement of a host factor [30].

The requirement of INI1 for HIV-1 late events was supported by additional lines of evidence, where it was demonstrated that the lack of INI1 in producer cells leads to the inhibition of HIV-1 particle production [15,27,29]. The expression of HIV-1 vectors in rhabdoid tumor-derived *INI1*^−/−^ MON cells led to decreased particle production, and the expression of INI1 complemented the defects in HIV-1 production in these cells [15,29]. Furthermore, INI1 mutants defective for binding to IN did not complement these defects in *INI1^−/−^* MON cells [32]. In addition, the shRNA-mediated knock-down of *INI1* in 293T cells led to defective particle production due to reduced trafficking of Gag and Gag-Pol to the membrane [27]. Additional studies have demonstrated that the presence of S6 causes a defect in early stages of assembly, where no budding virions were observed in the producer cells, despite the expression of Gag and GagPol [26]. Taken together, these studies suggested that a lack of INI1 or expression of the trans-dominant mutant S6 caused a reduction in HIV-1 particle production, which is in part due to the inhibition of Gag/GagPol trafficking and/or assembly.

Interestingly, while a lack of INI1, or the expression of the trans-dominant negative mutant S6, led to the inhibition of HIV-1 particle production, a different phenotype was observed when IN mutants were made defective for interaction with INI1 [31]. In one study, INI1-interaction-defective IN mutants were isolated using a reverse yeast two-hybrid system. Among several IN-mutants, those that lie on the surface of IN were selected further for study and were confirmed for their expression and specific interaction-defect with INI1 [31]. These INI1-interaction-defective (IID)-IN mutants were incorporated into the full-length molecular clone of HIV-1_NL4–3_ and further characterized for their effect on viral replication. These IID-IN mutants (e.g., D202G, Q137R) were defective for replication in a multiday replication assay. Furthermore, these mutant viruses did not show defects in viral protein expression levels, assembly, or particle production, but rather, the virions with IID-IN mutations exhibited defective particle morphology [31]. The defect in particle morphology varied from immature capsids to eccentric capsids [31]. These virions with malformed particles showed defects in infectivity in the target cells and were impaired in early and late reverse transcription and integration [31].

The above studies collectively indicate that INI1 influences two distinct stages of HIV-1 replication: (1) early stages of assembly, where lack of INI1 or expression of an INI1 trans-dominant negative mutant (S6) in the producer cells leads to inhibition of particle production, and (2) particle morphogenesis, where mutations in IN that make it defective for binding to INI1 leads to impairment of particle morphology without inhibiting particle production. Thus, when INI1 is interfered with in producer cells, it leads to particle production defects, and when IN is mutated such that it no longer binds to INI1, it leads to defects in particle morphogenesis. While these studies indicated the importance of IN/INI1 interaction for HIV-1 late events, until recently, a lack of INI1 structural information significantly limited our understanding of the mechanism of its action in HIV-1 replication. Recently, we solved the NMR structure of the IN-binding Rpt1 domain of INI1 and determined the structural basis of IN/INI1 interactions [32]. These studies have helped us close the knowledge gap by revealing an unprecedented mimicry of the INI1-Rpt1 domain to HIV-1 TAR RNA [32], which explains the phenotypic overlap of IN mutants defective for binding to INI1 and those defective for binding to viral RNA (see below).

### 2.3. Structure of the Rpt1 Domain of INI1 and Structural Modeling of IN-CTD/INI1-Rpt1 Interactions

The NMR structure of the fragment of INI1_183–265_ that contains the IN-binding Rpt1 domain, linker, and part of the Rpt2 [32] indicates that it is monomeric in solution and consists of a well-ordered region with ββαα topology (aa 183–248) and a disordered linker region (aa 249–265) (PDB ID 6AX5) [32]. A slightly longer fragment INI1_183–304_ (Rpt1 + linker + part of the Rpt2) that more strongly binds to IN was modeled based on the similarity in Rpt1 and Rpt2 and was computationally docked onto the NMR structure of the IN-CTD [PDB ID: 1QMC] using in-house docking software, MDockPP [32,60,61]. The docked complex with the lowest (best) score of ITScorePP [62,63] indicated that upon complex formation between IN-CTD and INI1-Rpt1_183–304_, a large (∼865.0 Å^2^) solvent-accessible surface was buried [32]. The exposed negatively charged residues from the α-1 helix of Rpt1 formed hydrogen bonding interactions with positively charged residues of IN-CTD. The region of the hydrophobic interactions between Rpt1 and IN-CTD was buried and encircled by residues forming the hydrogen-bonding network interactions.

The IN-CTD/INI1-Rpt1 structural model was validated by testing the interface IN and INI1 residue mutations for their ability to interact using GST pull-down and Alpha assays [32]. Furthermore, functional significance of the IN residues interacting with INI1 was indicated by previous reports. Substitution mutations of the IN interface residues affected viral replication. W235E and W235K, but not W235F, inhibited integration and viral replication, consistent with this residue being in the buried hydrophobic pocket of the IN-CTD/INI1-Rpt1 complex; R228A, K244A, K264A/K266A, and R269A/K273A were found to be defective for HIV-1 replication; and K244A, K264A/K266A, and R269A/K273A were shown to be defective for binding to viral RNA. Together, these results suggested that the IN-interface residues of IN-CTD/INI1-Rpt1_183–304_ complex are important for HIV-1 replication [32]. Table 1 below is a list of IN residues present at the interface of IN-CTD/INI1-Rpt1_183–304_ complex and the effect of substitution mutations of these residues for (i) interaction with INI1; (ii) interaction with viral RNA; (iii) effect on viral replication; and (iv) particle morphology, as reported in various studies.

### 2.4. Structural Mimicry Between INI1-Rpt1 and TAR RNA

During these analyses, it was noted that some of the IN/INI1 interface residues (K264, R269) were also important for IN binding to HIV-1 genomic RNA [17,32] (Table 1). Substitution mutations of these interface IN residues (R228, W235, K264, R269), affected IN binding to both INI1 and TAR RNA and led to defective particle morphogenesis [17,32]. Our previous studies have indicated that IID IN mutants also led to defects in particle morphogenesis [31]. Based on these observations, it was surmised that IN residues involved in binding to INI1 and TAR RNA could overlap, and that this overlap in binding might explain the similarity in phenotypes of RNA-binding and INI1-binding-defective IN mutants in inducing particle morphogenesis defects. The following experimental results established the similarity of INI1 and TAR RNA binding to IN.

(i) **TAR RNA and INI1_183–304_ bind to the same residues of IN:** A panel of IN-CTD substitution mutations that span the interface residues of the IN-CTD/INI1-Rpt1 complex were tested for their ability to interact with TAR RNA using a protein–RNA interaction Alpha assay. The interaction profiles of TAR RNA and INI1_183–304_ with IN-CTD mutants were identical, indicating that these molecules recognize the same residues of IN [32] (see Table 1).
(ii) **TAR RNA and INI1_183–304_ compete for binding to IN-CTD:** TAR RNA and INI1_183–304_ competed for binding to IN-CTD with similar IC_50_ values (IC_50_ ≈ 5 nM) in an Alpha assay [32]. Furthermore, the inhibition of the IN-CTD/INI1-Rpt1 interaction by TAR was specific, as a scrambled RNA or a different fragment of HIV-1 genomic RNA (nts 237–279) did not inhibit CTD/INI1_183–304_ binding [32]. Together, these results indicated that INI1 Rpt1 and TAR require the same surface of IN-CTD for binding.
(iii) **Structural similarity between INI1 Rpt1 and HIV-1 TAR RNA:** To understand this further, the complex between IN-CTD and TAR RNA was computationally modeled using MdockPP [32,60,61]. It was found that the same set of hydrophobic and positively charged IN-CTD residues is involved in interaction with both INI1-Rpt1 and TAR RNA, confirming the biochemical studies (Figure 1C, left panel). When the complexes of IN-CTD/INI1-Rpt1 were superimposed onto the complex of IN-CTD/TAR, INI1-Rpt1 and TAR overlapped with each other in three-dimensional space (Figure 1C right panel) [32]. A close examination of the Rpt1 NMR structures indicated that it has a string of surface-exposed, negatively charged residues that are positioned in a specific manner. An examination of the position of phosphate groups on TAR, which overlap with INI1-Rpt1 in the superimposed structure, indicated that these phosphate groups are positioned in a manner resembling the arrangement of the negatively charged residues on the INI1-Rpt1 surface in three-dimensional space [32]. These analyses indicated that TAR RNA and INI-Rpt1 have overall similar shape and electrostatic charge distribution on the surface, explaining how these two molecules could contact the same residues on the surface of IN-CTD. This is consistent with the similarity in binding of these two molecules to IN [32].

The above study, for the first time, suggested that the Rpt1 domain of INI1 and TAR RNA structurally mimic each other [32]. This mimicry explains the requirement of the same IN residues for binding to INI1-Rpt1 and TAR RNA and similar phenotypes of INI1- and TAR RNA-binding defective IN mutants on particle morphogenesis.

### 2.5. A Model to Explain the Role of INI1 in HIV-1 Late Events Based on Its RNA Mimicry

Mimicry of proteins by nucleic acids exists in nature [80,81,82]. But mimicry between INI1-Rpt1 and HIV-1 TAR is a novel observation. INI1 binds to IN within the context of Gag-Pol and is incorporated into the virions in an IN-dependent manner [15,30]. A lack of INI1 inhibits particle production, and IN mutations defective for binding to INI1 do not affect particle production but lead to defects in particle morphology. Based on these observations and structural mimicry of the INI1-Rpt1 domain to TAR RNA, a model has been proposed to explain the role of INI1 in facilitating HIV-1 assembly.

This model is based on the possibility that binding of viral RNA to IN within the context of Gag-Pol during assembly may pose steric constraints. The 3-dimensional positioning of Gag and Gag-Pol to generate a 3D virion bud from a 2D planar lipid bilayer is likely to require significant structural mobility of Gag, Gag-Pol, and RNA [37,83]. Viral RNA binding to both the NC and MA portions of Gag, as well as to the IN portion of Gag-Pol, may cause steric hindrance during this process and may impose difficulties during assembly. Since INI1-Rpt1 and TAR bind to the same IN surface and compete for binding to IN, this model posits that INI1 binding to IN prevents RNA from binding to IN during assembly to overcome this steric hindrance (Figure 1D, panel 1). Thus, INI1 may act as a “place-holder”, which would be critical for assembly, and a lack of INI1 would inhibit assembly, consistent with observations [15,27,29,30] (Figure 1D, panels 1 and 2). The place-holder function has been demonstrated for other RNA-mimicking proteins involved in RNP assembly in yeast [80,81,82]. This model also explains why there is no inhibition of particle production when there are IN mutants defective for binding to either INI1 or RNA, as steric hindrance would be relieved, allowing assembly and particle production (Figure 1D, panel 3). Thus, particles are produced when there is a mutation in IN that makes it defective for binding to RNA or INI1, but when there is wild-type Gag-Pol, INI1 is required for assembly. However, the binding of RNA (and/or INI1) to IN appears to be required for particle morphogenesis, which is a step after assembly, particle production, and proteolysis (Figure 1D, panel 3). The inability of IN to bind to RNA and/or INI1 leads to morphologically defective particles.

### 2.6. Role of RNA and/or INI1 in Particle Morphogenesis

Several questions remain. At this point, it is unclear why IN mutants defective for binding to RNA or INI1 are morphologically defective. Also, it is hard to distinguish if RNA, INI1, or both are required for particle morphogenesis, as the mutants that are defective for binding to one are also defective for binding to the other molecule. Studies of compensatory mutations of RNA binding IN mutants (R269A/K273A substitutions) indicated that charged residues of IN are important for its RNA binding [68]. These compensatory mutants also restored the defect of R269A/K273A mutants for particle morphogenesis. It is clear from these studies that RNA binding to charged residues of IN is important for morphogenesis. However, since RNA and INI1-Rpt1 mimic each other, it will be interesting to see if these compensatory mutations restore the binding to INI1 as well, which has yet to be tested.

At this point, it is not clear what role INI1 may play during particle morphogenesis, if at all, or if it is required for that function. Our previous report indicates that INI1 binds and recruits SAP18 and some of the components of the HDAC1 complex into virions during assembly [28]. The overexpression of catalytically inactive HDAC1^H141A^ mutant did not affect particle production, but the particles produced were defective for infection and for reverse transcription [28]. One intriguing possibility is that the SAP18 and HDAC1 complex associated with INI1 that is recruited into virions may assist in particle morphogenesis. However, it is unclear at this point if the particles produced in the presence of HDAC1^H141A^ are defective morphologically. More experiments are needed to understand the role of INI1-associated SAP18 and HDAC1 in HIV-1 particle morphogenesis and/or infectivity.

## 3. Discussion

INI1 is an IN-binding host factor, and it influences HIV-1 replication at multiple stages, including assembly, particle production, and morphogenesis. The interaction of INI1 with IN is mediated via the INI1-Rpt1 domain. Structural mimicry of INI1-Rpt1 and HIV-1 TAR RNA explains the dual phenotype observed for this host factor, as explained in the model (Figure 1D) [32]. The proposed working model is based on the known functional data available about INI1 influence on HIV-1 replication, the computational docking of known NMR structures of IN-CTD/INI1-Rpt1, and the biochemical and mutagenesis data. Currently, no structures are available for the full length or the domains of the IN:INI1 or IN:RNA complex. Furthermore, for the sake of simplicity, the model in Figure 1 indicates a 1:1 stoichiometric ratio between IN and INI1. However, the correct stoichiometric ratio of IN:INI1 has not been established. A rough estimate of the stoichiometric ratio of IN:INI1 in the virions indicated a 2:1 ratio, suggesting a dimer of IN may bind to a monomer of INI1 [30]. Structural and biochemical studies that inform the stoichiometric ratio of IN:INI1 and the details of the interactions of the two proteins are required to fully comprehend the mechanism.

It has been well established that the functional unit of IN is a tetramer [84]. IN-CTD exists as a dimer in native form, and the interface of CTD required for binding to RNA or INI1, based on our proposed model, would be occluded in this state [84]. However, recent structural studies suggest that CTDs can assume different conformations within a tetramer or higher-order structures [85]. In a tetrameric intasome structure, while the CTDs from inner protomers are engaged in interaction with DNA, the CTDs from the outer protomers are not [85]. Furthermore, a recent report of the native tetrameric structure of IN indicates that while the inner CTDs of the tetramers are arranged in an interlocking position and are engaged in interaction with other domains of IN, a patch of positively charged residues of the outer CTDss are available for interaction with vRNA [86]. These studies suggest that in a tetramer or a higher-order structure of IN, while the CTD from inner protomers could engage in interaction with other domains of IN or DNA, the CTDs from outer protomers are available for interaction with INI1 or RNA.

Our model, which is based on the “place holder” function of other known RNA-mimicking proteins, proposes that INI1 and vRNA binding to IN is spatially and temporally separated. We propose that INI1 binds to IN within the context of GagPol during assembly in the cells and that INI1 is replaced by vRNA in the mature virion. At this point, it is not known how the temporal replacement of INI1 by RNA takes place in the mature virion. One possibility is that differences in the multimeric nature (dimer, tetramer, or higher-order structure) and/or conformations of IN within GagPol versus processed IN could determine which of the two molecules, INI1 or RNA, binds to IN [85,86,87]. Determining the structure of GagPol and IN bound to RNA or INI1 would shed light on these important questions. Furthermore, future isolation and characterization of IN mutations that differentially affect the binding of INI1 and RNA are required to shed light on this model and to provide insight about the specific roles of RNA and INI1 in particle morphogenesis.

Considering that INI1 is a component of the SWI/SNF chromatin remodeling complex, it plays an additional role during HIV-1 replication. It has been established that the SWI/SNF complex and INI1 are recruited to the LTR promoter and regulate transcription of the provirus [88,89,90,91]. This property of INI1 as a part of the SWI/SNF complex may also facilitate integration and targeting of the provirus [21,47]. In vitro studies have indicated that INI1 and the SWI/SNF complex facilitate HIV-1 integration into chromatin [47], while other studies have suggested that the INI1 fragment inhibits integration in vitro [92]. Our study has indicated that INI1 stimulates or inhibits integration depending on the concentration of IN [21,52]. While INI1 stimulates integration at lower concentrations of IN, it inhibits integration at higher concentrations of IN. These are in vitro studies and have not been substantiated in cells. Finally, it was suggested in one study that INI1 was antiviral, based on the stimulatory effects on early events shown by IN mutant K71R, which was partially defective for interaction with INI1 [93]. However, another study indicated that viruses harboring K71R were partially defective for replication [31]. It is intriguing to note that INI1 is necessary for the induction of interferon signaling, which makes it an antiviral host factor [57]. More studies are needed to understand how the functions of INI1 in inducing interferon signaling influence HIV-1 replication.

In summary, the current available knowledge about INI1/IN interaction and influence of INI1 on HIV-1 replication paints an incomplete yet rich and complex host–virus interplay. Future inquiry into this interplay is required, which will likely open up new lines of investigation into the biology of HIV-1 and the host factor. The questions that need to be addressed include, but are not limited to, the following: (1) How and when during HIV-1 replication does INI1 dissociate from SWI/SNF complex, get exported into the cytoplasm, and associate with IN to be incorporated into virions? If INI1 is shuttling between the nucleus and the cytoplasm, is it possible that binding to GagPol retains it in the cytoplasm and recruits it into the virions? (2) What is the stoichiometry of IN:INI1, and what determines the association of INI1 versus vRNA with GagPol or IN? Does the multimeric nature of GagPol or IN determine the spatial and temporal association of INI1 and RNA? (3) What is the mechanism by which IN facilitates proper morphogenesis of the virion particles, via. its binding to vRNA or INI1? (4) Since INI1 is part of the chromatin remodeling SWI/SNF complex, is INI1 in the virions required for the integration of the viral DNA and/or for the subsequent transcription of the integrated proviral DNA?

The interaction of INI1 with HIV-1 IN and Gag-Pol, its requirement for late events, and its RNA mimicry of viral TAR RNA in binding to IN make it an outstanding candidate for developing antivirals to inhibit late events. The structural information of the INI1-Rpt1 domain and IN-CTD/INI1-Rpt1 interaction and the newly discovered RNA mimicry of INI1 establish the IN/INI1 interface as a promising drug target and provide insights into the development of novel anti-HIV strategies. Protein-protein interactions (PPI) between host and viral proteins are valuable targets to inhibit viral replication. While large and flat interacting surfaces often preclude the use of small molecules as drugs to disrupt PPI, larger biologics such as peptidomimetics (e.g., hydrocarbon-stapled peptide mimetics) are promising inhibitors of the PPIs that were previously intractable [94,95]. Such biologics that disrupt IN/INI1 interactions are likely to also inhibit IN/RNA interactions, making them attractive dual-acting inhibitors for future drug development. These biologics will not only be beneficial for anti-retroviral therapy, likely with a lower propensity to elicit drug resistance, but they may also be valuable for understanding the role of INI1, IN, and Gag-Pol in assembly, particle production, and particle morphogenesis.

## Figures and Tables

**Table 1:** List of IN-CTD residues at the interface contacting INI1-Rpt1 residues and the effect of substitution mutations of these residues.

IN Residues	IN Mutations *, **	IN-INI1 Interaction	IN-RNA Interaction	Infection	Capsid Morphology	Reference
**Charged**						
**R228**	**R228A**	**Defective**	**Defective**	**Defective**	**Defective**	[11,32,64]
**K244**	**K244A**	**Defective**	**Defective**	**Defective**	**ND**	[32,64]
	K244E	ND	ND	Defective	ND	[65]
	K244A/E246A	ND	ND	Defective	ND	[66]
	K240A, K244A/R263A, K264A	ND	ND	Defective	ND	[67]
**R262**	R262A	ND	ND	Not Defective	ND	[64]
	**R262A/R263A**	**ND**	**Defective**	**Defective**	**Defective**	[11,64,68]
	R262A/K264A	ND	ND	Defective	ND	[64]
	R262I/K264T	ND	ND	Defective	ND	[69]
	R262D/R263V/K264E	ND	ND	Defective	ND	[65]
**R263**	R263A	ND	ND	Less Defective	ND	[64]
	R263K	ND	ND	Not Defective	ND	[70]
	R263L	ND	ND	Not Defective	ND	[65]
	R263S	ND	ND	Not Defective	ND	[69]
	R263A/K264A	ND	ND	Defective	ND	[71]
**K264**	K264A	ND	ND	Not Defective	ND	[64]
	K264E	ND	ND	Defective	ND	[64]
	K264R	ND	ND	Not Defective	ND	[72]
	**K264A/K266A**	**Defective**	**Defective**	**Defective**	**Defective**	[17,32]
	K264R/K266R/K273R	ND	ND	Defective	ND	[73]
**R269**	R269A	ND	ND	Reduced and delayed	ND	[64,65]
	R269A/D270A	ND	ND	Reduced	ND	[64,65]
	**R269A/K273A**	**Defective**	**Defective**	**Defective**	**Defective**	[17,32,74]
**Hydrophobic**						
**I220**	I220L	ND	ND	Slightly Reduced	ND	[75]
**F223**	F223A	ND	ND	Defective	ND	[76]
	F223E	ND	ND	Defective	ND	[76]
	F223G	ND	ND	Defective	ND	[76]
	F223H	ND	ND	Less Defective	ND	[76]
	F223K	ND	ND	Defective	ND	[76]
	F223S	ND	ND	Defective	ND	[76]
	F223Y	ND	ND	Not Defective	ND	[76]
**W235**	**W235A**	**Defective**	**Defective**	**Defective**	**ND**	[32,34,77]
	**W235E**	**Defective**	**Defective**	**Defective**	**Defective**	[32,34,77]
	**W235K**	**Defective**	**Defective**	**Defective**	**ND**	[32,34,77]
	**W235F**	**Not Defective**	**Not Defective**	**Not Defective**	**ND**	[32,34,77]
**A265**	A265T	ND	ND	Not Defective	ND	[78]
	A265V	ND	ND	Not Defective	ND	[78,79]

ND = not determined. * Mutations that have been characterized for all four functions (interaction with INI1, with RNA, viral infection, and particle morphology are highlighted with a gray box. ** Mutations that have been characterized for at least three of the four above functions are bolded.

## Abbreviations

The following abbreviations are used in this manuscript:

2D	Two-dimensional
3D	Three-dimensional
aa	Amino acid
AIDS	Acquired immunodeficiency syndrome
ALLINI	Allosteric inhibitors of integrase
ART	Anti-retroviral therapy
ATP	Adenosine triphosphate
BAF47	Bramha-related gene (BRG)1-associated factor 47
CA	Capsid
cDNA	Complementary deoxyribonucleic acid
CTD	C-terminal domain
DBD	DNA-binding domain
DNA	Deoxyribonucleic acid
GST	Glutathione S-transferase
HADDOCK	High ambiguity driven protein–protein docking
HDAC1	Histone deacetylase 1
HIV	Human immunodeficiency virus
HR3	Homology region III
hSNF5	Human sucrose non-fermenting
IBD	Integrase-binding domain
IC_50_	Half-maximal inhibitory concentration
IID	INI1-interaction-defective
IN	Integrase
INI1	Integrase interactor 1
LEDGF	Lens epithelium–derived growth factor
LTR	Long terminal repeat
MA	Matrix
NC	Nucleocapsid
ND	Not determined
NES	Nuclear export signal
NMR	Nuclear magnetic resonance
nts	Nucleotides
PDB	Protein Data Bank
PPI	Protein–protein interaction
PR	Protease
RNA	Ribonucleic acid
RNP	Ribonucleoprotein
Rpt1	Repeat 1
Rpt2	Repeat 2
RT	Reverse transcriptase
SAP18	Sin3A associated protein 18
shRNA	Short hairpin ribonucleic acid
SIV	Simian immunodeficiency virus
SMARCB1	SWI/SNF-related matrix-associated actin-dependent regulator of chromatin subfamily B member 1
SWI/SNF	Switch/sucrose non-fermenting
TAR	Trans-activation response
Tat	Trans-activator of transcription
Vpr	Viral protein R
WHD	Winged-Helix DNA-binding domain
WT	Wild-type
